# Editorial: New insights into the role of complement system in liver diseases

**DOI:** 10.3389/fimmu.2023.1284944

**Published:** 2023-09-08

**Authors:** Zhenya Guo, Xiude Fan, Laura E. Nagy, Stephen Tomlinson, Guandou Yuan

**Affiliations:** ^1^ Division of Hepatobiliary Surgery, the First Affiliated Hospital of Guangxi Medical University, Nanning, Guangxi, China; ^2^ Key Laboratory of Early Prevention and Treatment for Regional High Frequency Tumor (Guangxi Medical University), Ministry of Education, Nanning, Guangxi, China; ^3^ Guangxi Key Laboratory of Immunology and Metabolism for Liver Diseases, Guangxi Medical University, Nanning, Guangxi, China; ^4^ Department of Endocrinology, Shandong Provincial Hospital Affiliated to Shandong First Medical University, Jinan, Shandong, China; ^5^ Northern Ohio Alcohol Center, Department of Inflammation and Immunity, Cleveland Clinic, Cleveland, OH, United States; ^6^ Department of Microbiology and Immunology, Medical University of South Carolina, Charleston, SC, United States

**Keywords:** complement, nonalcoholic fatty liver disease, hepatocellular carcinoma, ischemia-reperfusion injury, antibody-mediated rejection

The complement system is a highly conserved and evolutionary ancient arm of immunity, and it plays a key role in immune surveillance and tissue homeostasis. The complement system comprises an extensive network of fluid-phase and membrane bound glycoproteins, cofactors, receptors and regulatory proteins, and comprises of more than 50 components; it engages in innate immune recognition and inflammatory effector responses, as well as in modulating adaptive immune responses. The liver is the main source of serum complement proteins, and there are multiple complement receptors expressed on parenchymal cells, as well as non-parenchymal cells such as Kupffer cells and hepatic stellate cells. The complement system is known to have an essential role in maintaining the immune microenvironment within the liver. While there are clear correlations between complement activation and various liver diseases, in many cases the underlying mechanisms involved in the propagation of liver pathologies remains elusive. In this Research Topic, we aim to bring new insights to:

1. The complement system and nonalcoholic fatty liver disease (NAFLD).
2. The complement system and hepatic ischemia-reperfusion injury (IRI).
3. The complement system and hepatocellular carcinoma (HCC).
4. The complement system and antibody-mediated rejection (AMR) in liver transplantation.

## Complement system and NAFLD

The complement system is known to play a role in the pathological mechanism of NAFLD. In order to evaluate the association of complement components with the risk and severity of NAFLD, Zhao et al. performed a meta-analysis involving 18 studies with 18560 subjects. They found that the complement proteins C3, C5, factor B and acylation stimulating protein are associated with an increased risk and severity of NAFLD. This study suggests that certain complement proteins may be potential therapeutic targets as well as diagnostic biomarkers of NAFLD. Guo et al. review current research on complement-mediated mechanisms involved with NAFLD. In this review, Guo et al. provide an integrative picture of the role of complement in the progression of NAFLD. In early stages of NAFLD, aberrant complement activation is involved in insulin resistance and lipid metabolism disorder, which leads to hepatocyte steatosis. In the late stage, complement contributes to nonalcoholic steatohepatitis by regulating the abundance and function of immune cells, such as macrophages, neutrophils and T cells.

Although complement was long regarded as a systemic serum-based component of immunity, it is now clear that the complement system is also active intracellularly. The article by Xiao F. et al. review what is known about this intracellularly active complement system, which is known as “the complosome”. Discussed is the important role of the complosome in metabolism and immune responses.

## Complement system and IRI

IRI is an unavoidable consequence of many liver surgeries, and it often leads to severe complications. In IRI, complement activation products contribute to the recruitment and activation of macrophages and neutrophils which cause oxidative stress and inflammation. The terminal complement activation product, the cytolytic membrane attack complex, has also been shown to play a central role in hepatic IRI (1). Numerous studies indicate that complement inhibition represents a potential therapeutic strategy to protect against hepatic IRI. However, some complement activation products are also essential for liver regeneration, such as after partial hepatectomy (2). Using a mouse model, Kusakabe et al. find that Properdin may be an ideal therapeutic target. Properdin is the only known positive regulator of alternative pathway, a pathway that also functions as an amplification loop for the classical and lectin activation pathways (3). In this study, Kusakabe et al. show that an anti-properdin antibody significantly attenuates hepatic IRI without compromising liver regeneration. This work suggests that targeting properdin may be an optimal therapeutic approach for surgeries involving liver resection and liver transplantation.

## Complement system and HCC

HCC is the most common type of liver cancer and is a global health burden with limited treatment options and poor prognoses. Recent studies investigating strategies of immune checkpoint inhibition have yielded some remarkable results, and immunotherapeutic approaches hold great hope for curing cancer (4). The complement system, as a key system for immune surveillance and homeostasis, has been shown to play a role in HCC. Xiao Z. et al. review recent findings on the role of complement in HCC. In their review, Xiao Z. et al. craft a schematic to illustrate the intricate interactions between complement and HCC. Findings show that complement plays a dual role in HCC; for example, C1q, C3/C3a and C5/C5a are upregulated in HCC and are associated with aggressive tumor phenotypes. On the other hand, C2, C6, C8 and mannose-binding lectin are downregulated in HCC and exhibit a tumor-suppressive effect. These molecules could represent therapeutic targets or prognostic markers for HCC. To this end, Xiao Z. et al. ‘s review summarizes current understanding of the association between complement and HCC, and how this understanding may help with the development of novel therapeutic complement-targeted strategies for HCC.

## Complement system and AMR

AMR following liver transplantation remains a refractory humoral rejection threatening graft and patient survival (5). Currently, there is lack of an effective treatment, and furthermore there is lack of an adequate animal models for developing or validating new interventions. Tajima et al. report on the establishment of a rat AMR model in the context of orthotopic liver transplantation. In order to sensitize B cells effectively, Tajima et al. creatively utilize skin transplantion for pre-sensitization. They show that an anti-C5 antibody effectively ameliorates AMR of liver grafts, suggesting that complement-targeted therapies may be a promising strategy for treating AMR.

In summary, the contributions in this Frontiers TOPIC highlight promising aspects of complement research as it relates to developing new therapeutic strategies for liver disease.

## Author contributions

ZG: Conceptualization, Writing – original draft. XF: Conceptualization, Supervision, Writing – review & editing. LN: Conceptualization, Supervision, Writing – review & editing. ST: Conceptualization, Supervision, Writing – review & editing. GY: Conceptualization, Supervision, Writing – original draft, Writing – review & editing.
